# Electro-acupuncture decreases 5-HT, CGRP and increases NPY in the brain-gut axis in two rat models of Diarrhea-predominant irritable bowel syndrome(D-IBS)

**DOI:** 10.1186/s12906-015-0863-5

**Published:** 2015-09-29

**Authors:** Jianhua Sun, Xiaoliang Wu, Yunfang Meng, Jie Cheng, Houxu Ning, Yongjun Peng, Lixia Pei, Wei Zhang

**Affiliations:** Department of Acupuncture and Rehabilitation, Affiliated Hospital of Nanjing University of Chinese Medicine, Nanjing, 210029 Jiangsu Province China; Shanghai Key Laboratory of Molecular Mycology, Department of Dermatology, Changzheng Hospital, Second Military Medical University, Shanghai, 200003 China; Nanjing University of Chinese Medicine, Nanjing, 210029 Jiangsu Province China; Nanjing Brain Hospital Affiliated to Nanjing Medical University, Nanjing, 210029 Jiangsu Province China; Chinese Medicine Hospital of Jiangsu Province, 155 Hanzhong Road, Nanjing, 210029 Jiangsu Province China

**Keywords:** Diarrhea-predominant irritable bowel syndrome (D-IBS), Brain-gut axis (BGA), Electro-acupuncture (EA), 5-hydroxytryptamine (5-HT), Calcitonin gene-related peptide (CGRP), Neuro-peptide Y (NPY)

## Abstract

**Background:**

To examine whether electro-acupuncture (EA) could decrease 5-hydroxytryptamine (5-HT) and calcitonin gene-related peptide (CGRP), and increase neuro-peptide Y (NPY) in the brain-gut axis (BGA) in D-IBS using rat models.

**Methods:**

Rats were randomly exposed to unpredictable chronic stress for 3 weeks followed by 1-hour acute restraint stress (CAS) after 7 days of rest, or daily gavage of Senna decoction (6 g/kg) plus chronic restraint stress (for a duration of 2 h, starting from 1 h prior to the gavage) for 2 weeks (ISC). The content of 5-HT, CGRP and NPY in the distal colon, spinal cord, hypothalamus was examined at the end of the treatment.

**Results:**

1. The two rat models exhibited similar characteristics, e.g., increased number of fecal pellets expelled in 1 h, decreased sacchar-intake, decreased CRD, elevated 5-HT, CGRP content and decreased NPY in the distal colon, spinal cord, hypothalamus (P < 0.05 vs. that in healthy control rats). 2. A series of equations was developed based on correlation regression analysis. The analysis results demonstrated that 5-HT mediates the changes in hypothalamus, spinal cord and colon. 5-HT and CGRP in spinal cord was closely correlated with general behavior evaluation and other transmitters in BGA.

**Conclusion:**

1. In comparison to 5-HT, CGRP and NPY (particularly in the spinal cord) had closer relationship with the D-IBS symptoms induced by either stress factors or Senna decotion.

2. EA treatment could restore the brain-gut axis to balanced levels.

## Background/Introduction

Diarrhea-predominant irritable bowel syndrome (D-IBS) is one of the most common subtypes of IBS. The causes may include stress (both psychological and social) and dietary factors. Medications that alter the function of the gastrointestinal (GI) tract may also cause D-IBS. The symptoms of the disorder aggravate the stress, thus forming a vicious cycle. Many types of managements (including medications) are available to treat this disease, but all have limited efficacy.

A previous study from this research group [1] showed that electro-acupuncture (EA) could lower the severity and frequency of abdominal pain, diarrhea, abdominal distension, and increase the quality of life (QOL) in patients with IBS. Besides, acupuncture therapy also showed good regulation on the important influencing factors of IBS, as anxiety, conflict behavior, dietary restriction and social response, etc. Some relevant experiments and study about D-IBS found that acupuncture could regulate central nervous system, immune system, endocrine system and GI tract, particularly the excitability of brain functional areas and dorsale medullae spinalis neurons [2]. So is the effect of acupuncture on IBS just placebo effect, or real curative effect such as alleviation of the intestinal tract symptoms, regulation of psychological symptoms? Are there any differences of the effects on model rats between the inducements of stress factor and food-medicine factor? Are there any congenerous paths on mechanism and what’s their correlation?

Most existing methods of making model are trying to combine more than two stimulating methods in order to simulate IBS’s onset characteristics comprehensively but there is still no affirmative conclusion. We combined kinds of accepted stimulating methods and simulated two important inducements of IBS’s onset characteristics, stress factor and food-medicine factor. In the current study, we used two rat models of D-IBS to examine the potential efficacy of EA. Animal behaviors that reflect the disease phenotype and neurotransmitters (5-HT, CGRP, NPY) in the brain-gut axis (BGA) were examined. We made up two kinds of models, and tried to analyze the mechanism of IBS model rats’ BGA system modification by two stimulating methods and the effectiveness of EA interference on IBS.

## Methods

### Ethics statement

All procedures had the approval of the Animal Ethics Committee of the Nanjing University of Chinese Medicine.

### D-IBS and treatment

Wistar rats (100 ~ 120 g, four weeks of age, consisting of both genders at a ratio of 1:1) were housed individually in a specific-pathogen-free facility (22 ± 2 °C), under a 12/12 h light/dark cycle (lights on from 6:00 am). Standard rat food and tap water were available without restriction unless otherwise noted. After 1-week acclimation, rats were randomly exposed to one of the following conditions: unpredictable chronic stress for 3 weeks followed by 7 days of rest and then 1-h acute restraint stress (rat model of chronic and acute stress, CAS) [3], or daily gavage of senna decoction (6 g/kg) plus chronic restraint stress (for a duration of 2 h, starting from 1 h prior to the gavage) for 2 weeks (rat model of senna gavage and chronic, ISC) [4]. Food (but not water) was removed 10 h prior to the exposure. The unpredictable chronic stress included the following seven stressors at random order: overnight illumination for 12 h, 45 °C environment for 5 min, water deprivation for 24 h, 4 °C environment for 3 min, tail clamp for 1 min, level vibration (120/min) for 40 min, and food deprivation for 24 h. Behavior testing was conducted between 8.00 am and 12.00 am. Defecation was examined by counting the number of fecal pellets in a period of 1 h. Sacchar (1 % sucrose) intake and abdominal withdrawal reflex (AWR) were also examined at the end of the experiments.

Part of the rats received EA treatment (15-min daily session for a duration of 2 weeks), starting from the second day after the exposure, using a paradigm described below.

Rats were immobilized using a homemade board. EA was delivered to the following sites bilaterally at a depth of 3-mm under aseptic condition: ST25 (tianshu), ST36 (zusanli), and LR3 (taichong), using disposable Huatuo acupuncture needles for cosmetic use (0.19 mm × 10 mm; Suzhou Medical Appliance Factory; Suzhou, China) [5]. EA was delivered using a stimulator for 15 min (HANS-200A, Nanjing Jisheng Medical Technology Co., Ltd., China) in alternating trains of dense-sparse rectangular stimuli (2/15 Hz, 0.8 ~ 1.3 mA).

Colonic motility was examined by counting the number of fecal pellets for a period of 1 h. Sacchar (1 % sucrose) intake was used to reflect anhedonia, as described previously. AWR was examined in response to colorectal distention (CRD), as explained below. A polyethelene balloon (length: 1.5 cm; diameter: 0.9 cm) was inserted into the descending colon. The rat was placed on a 20 × 8 × 8 cm platform and allowed to adapt for 15 min. Measurement with each fluid volume in the balloon to induce AWR lasted 10 s, and was repeated for three times, with 10-min interval.

A separate group of rats were included as a healthy control. The number of the animal subjects was 12–16 per group.

- Group 1: CAS model of D-IBS Without EA;
- Group 2: CAS model of D-IBS With EA;
- Group 3: ISC model of D-IBS Without EA;
- Group 4: ISC model of D-IBS With EA;
- Group 5: Heathy Control.
- Measurement of 5-HT, CGRP, and NPY

Rats were sacrificed under deep anesthesia with urethane (6 ml/kg, ip); the hypothalamus, spinal cord (lumbar intumescentia, L4-L6), and distal colon (at 8-cm proximal to colorectal junction) were excised and stored at −80 °C for analysis of 5-HT, CGRP and NPY content using enzyme linked immunosorbentassay (ELISA; all from R&D rat 5-Hydroxytryptamine (5-HT) & Calcitonin Gene Related Peptide (CGRP) & Neuropeptide Y (NPY) elisa kits provided by Nanjing jiancheng bioengineering institute). Distal colon was also fixed in neutral formalin for paraffin embedding, followed by HE staining and microscopic examination.

## Data analysis

One-way ANOVA or Kruskal Wallis Tests among groups was used to interpret the data for significant differences, the data were expressed as mean ± Standard deviation. Pearson’s correlation test was used to analyze the potential correlation between 5-HT, CGRP and NPY content in the hypothalamus, spinal cord and distal colon and behavioral measures, such as colonic motility, psychological ethology and colonic sensation. All analyses were carried out using SPSS version 17.0 (SPSS, Chicago, IL, USA). Statistically significant differences were defined at P < 0.05.

## Results

Five rats (one in CAS rats with EA, two each in CAS and ISC rats without EA) developed macro- or micro- intestinal bleeding, and were not included in data analysis. Otherwise, microscopic examination revealed healthy colon mucous membrane structure, including cryptae, epidermis and all other tissues.

1. Comparison between groups

Because the data of the five groups didn’t accord with normal distribution and homogeneity of variance, we chose the statistic method of Kruskal Wallis Test to explore the comparison of general behavioral evaluation distribution between groups.

**Fig. 1:**
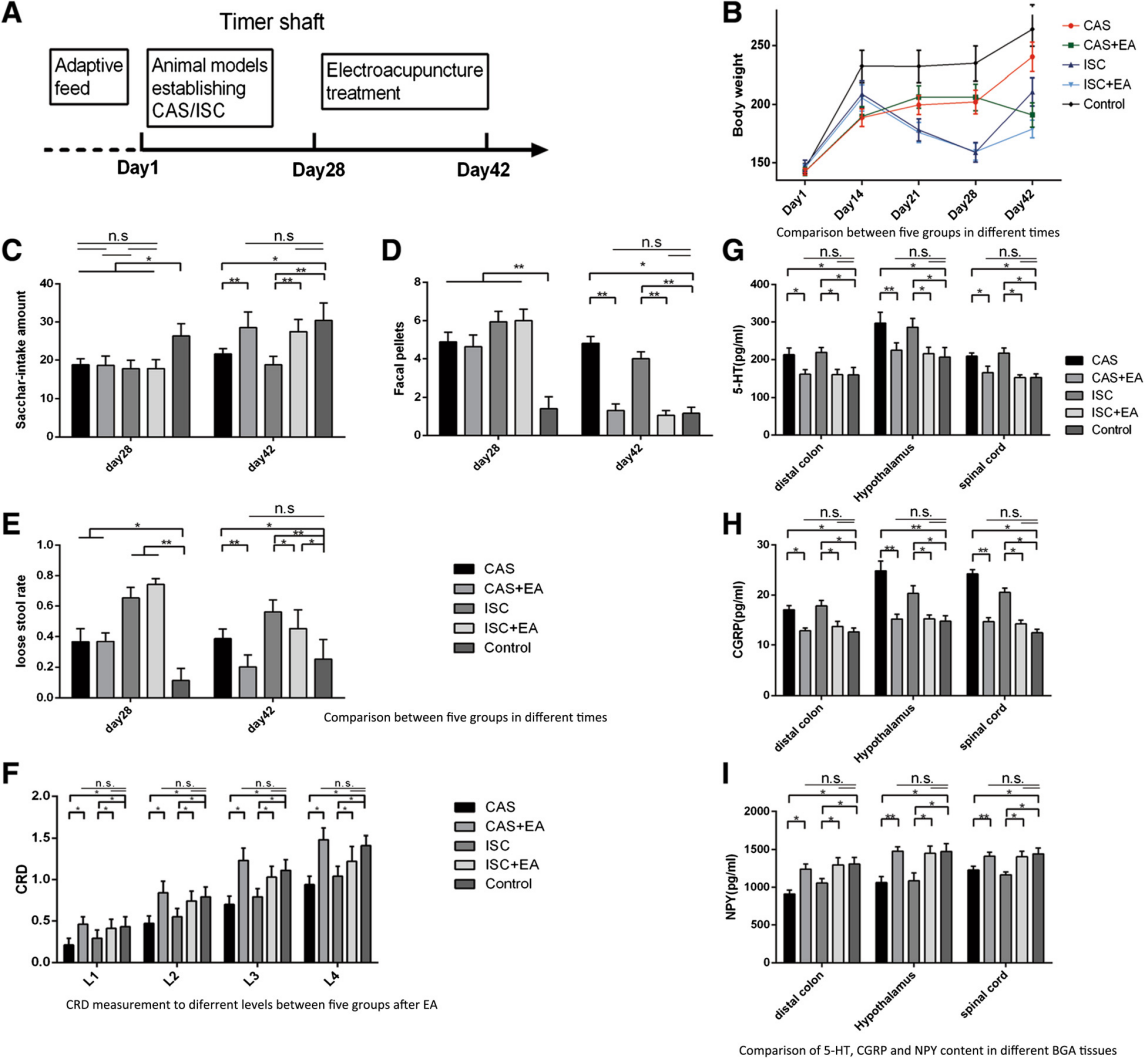
Comparison of general bahavioral evaluation distribution (mean±SD)

Body weight, fecal pellet number, loose stool rate and sacchar-intake at baseline did not differ between the two paradigms (P > 0.05; Fig. 1a, b, c, d, e). Starting from one week after its implementation, chronic stress decreased the body weight (P < 0.05 between CAS and ISC paradigms). EA decreased the body weight significantly (P < 0.05).

Fecal pellet number was increased by chronic stress (P < 0.05 vs. the healthy control, Fig. 1d). The EA treatment decreased the fecal pellet number under both conditions to a level comparable to the healthy control (P > 0.05 vs. the healthy control). EA decreased the number of fecal pellets in CAS rats and ISC rats.

Effects of the behavioral paradigms and EA on loose stool rate were generally similar to that in the number of fecal pellets. However, the loose stool rate was higher in ISC rats, with or without EA, in comparison to that in CAS rats, indicating that Senna leaf could induce loose stool (Fig. 1e).

The stress decreased sacchar-intake in CAS rats and ISC rats (P < 0.05 vs. healthy control). EA treatment incerased sacchar-intake to a level comparable to the healthy control (P > 0.05 vs. the healthy control) (Fig. 1c). Both the CAS group and ISC group have a lower CRD value (P < 0.05 vs. healthy control), and EA treatment increased CRD (P < 0.05 vs. CAS group and ISC without EA) to a level comparable to that in the healthy control (Fig. 1f).

The CAS and ISC rats had higher 5-HT, CGRP content and lower NPY content in the colon, the spinal cord, the hypothalamus (P < 0.05 vs. healthy control). EA decreased 5-HT and CGRP content in the distal colon, spinal cord, hypothalamus in both CAS and ISC rats (P < 0.05, vs. CAS group and ISC without EA). NPY content was increased by EA (P < 0.05, vs. CAS group and ISC without EA) to levels comparable to that in the healthy control rats (P > 0.05, vs. healthy control). EA treatment reduced 5-HT, CGRP content and increased NPY content in both CAS and ISC rats (Fig. 1g, h, i).

2. Analysis of the coefficient correlation between factors

Body weight was higher in male rats. Sacchar-intake correlated negatively with the number of fecal pellets (r = −0.694) and CGRP content of the spinal cord (r = −0.668).

A regression analysis revealed the following equation: CRD = 0.095 + 0.285 X, in which X is AWR level (1–4) (R = 0.837, r^2^ = 0.700). The adjusted coefficient of determination (r^2^) was 0.699. T was 3.032 and 24.899 and P was 0.003 and <0.001, respectively upon *t*-test (P < 0.05).

5-HT content in distal colon, spinal cord, hypothalamus could be described as Y = 54.683 + 0.216X_1_ + 0.416X_2_, in which X_1_ is 5-HT content of hypothalamus, and *X*_2_ is 5-HT content of spinal cord. The coefficient of regression of X_1_ and *X*_2_ is 0.216 and 0.416, respectively (Table 1). T was 2.242, 2.899 and 3.077 respectively, whereas P was 0.028, 0.005 and 0.003, respectively upon *t*-test. R = 0.566, r^2^ = 0.321 and the coefficient of determination was 0.299. NPY content of colon was proportional to CGRP content of colon and spinal cord (R = 0.652, r^2^ = 0.425, R^2^ = 0.397), and could be described using the following equation: Y = 1240.389 + 45.471X_1_-37.872X_2_, in which the NPY content of colon and X_1_ and *X*_2_ are CGRP content of colon and spinal cord respectively. The regression coefficient was 45.471 and −37.872, respectively. T was 9.623, 4.890 and −5.183 whereas P was <0.001 for all. The exclusion coefficient was 0. T value for regression between the CGRP content of hypothalamus and the NPY content of colon was −0.872 (P = 0.387). The possibility of T at 0 could not be excluded. Other regression equations (Table 1) are in accordance with the above analysis.

**Table 1:** Analysis of the correlation coefficient between various factors and neuropeptides

Dependent Variables(Y)	Predictors: (Constant)(X)	R	R Square	Adjusted R Square	Linear Regression(Y = B_0_ + B_1_X_1_ + B_2_X_2_ + … + B_n_X_n_)	Model	B	Std. Error	Beta	t	Sig.
body weight	NPY in the colon, hypothalamus, spinal cord	Excluded									>0.05
body weight	5-HT in the colon, hypothalamus, spinal cord	Excluded									>0.05
body weight	CGRP in the colon, hypothalamus, spinal cord	Excluded									>0.05
fecal pellets at 1 h	X is CGRP in spinal cord	0.666	0.444	0.417	Y = −2.916 + 0.140X	(Constant)	−2.916	0.836		−3.489	0.001
						CGRP of spinal cord	0.14	0.047	0.361	2.96	0.004
	Excluded Variables: CGRP in the colon, hypothalamus					CGRP of colon	0.105	0.06	0.195	1.749	0.085
						CGRP of hypothalamus	0.076	0.04	0.239	1.92	0.059
fecal pellets at 1 h	X_1_ is 5-HT of colon, *X*_2_ is 5-HT of spinal cord	0.588	0.345	0.325	Y = −2.005 + 0.013X_1_ + 0.012X_2_	(Constant)	−2.005	0.806		−2.487	0.016
						5-HT of colon	0.013	0.004	0.377	3.271	0.002
	Excluded Variables: 5-HT of hypothalamus					5-HT of spinal cord	0.012	0.005	0.304	2.633	0.011
fecal pellets at 1 h	X is NPY of spinal cord	0.568	0.322	0.29	Y = 9.619-0.003X	(Constant)	9.619	1.332		7.221	<0.0001
						NPY of spinal cord	−0.003	0.001	−0.408	−3.762	<0.0001
	Excluded Variables: NPY of colon, hypothalamus					NPY of colon	−0.001	0.001	−0.166	−1.421	0.16
						NPY of hypothalamus	0	0.001	−0.18	−1.547	0.127
fecal pellets at 1 h	X_1_is CGRP of spinal cord, *X*_2_ is NPY of spinal cord	STEP EXCLUDED			Y = 3.367 + 0.202X_1_-0.003X_2_	(Constant)	3.367	1.289		2.613	0.011
						CGRP of spinal cord	0.202	0.035	0.521	5.809	<0.0001
						NPY of spinal cord	−0.003	0.001	−0.391	−4.363	<0.0001
	Excluded Variables: 5-HT of colon, spinal cord, NPY of spinal cord					5-HT of spinal cord	.258a	2.349	0.022	0.282	0.769
						5-HT of colon	.392a	4.267	0	0.471	0.928
						NPY of spinal cord	-.391a	−4.363	0	−0.479	0.961
sacchar-intake amount	X is CGRP of spinal cord	0.682	0.466	0.44	Y = 39.289-0.635X	(Constant)	39.289	2.195		17.903	<0.0001
						CGRP of spinal cord	−0.635	0.124	−0.61	−5.105	<0.0001
	Excluded Variables: CGRP of colon, hypothalamus					CGRP of colon	−0.234	0.158	−0.162	−1.478	0.144
						CGRP of hypothalamus	0.024	0.104	0.029	0.235	0.815
sacchar-intake amount	X is 5-HT of spinal cord	0.418	0.175	0.162	Y = 33.278-0.045X	(Constant)	33.278	2.229		14.929	<0.0001
	Excluded Variables: 5-HT of colon, hypothalamus					5-HT of spinal cord	−0.045	0.012	−0.418	−3.711	<0.0001
sacchar-intake amount	X_1_ is NPY of colon, *X*_2_ is NPY of hypothalamus	0.541	0.292	0.259	Y = 9.175 + 0.005X_1_ + 0.004X_2_	(Constant)	9.175	3.648		2.515	0.014
						NPY of colon	0.005	0.002	0.279	2.336	0.023
	Excluded Variables: NPY of spinal cord					NPY of hypothalamus	0.004	0.002	0.271	2.275	0.026
						NPY of spinal cord	0.004	0.003	0.171	1.544	0.128
sacchar-intake amount	X is CGRP of spinal cord	STEP EXCLUDED			Y = 37.323-0.696X	(Constant)	37.323	1.731		21.567	<0.0001
						CGRP of spinal cord	−0.696	0.096	−0.668	−7.245	<0.0001
	Excluded Variables: 5-HT of spinal cord, NPY of hypothalamus, colon					5-HT of spinal cord	–.126a	−1.2	0.234	−0.148	0.769
						NPY of hypothalamus	.136a	1.285	0.203	0.159	0.755
						NPY of colon	.173a	1.701	0.094	0.208	0.798
AWR1	X is CGRP of spinal cord	0.556	0.309	0.299	Y = 0.624-0.015X	(Constant)	0.624	0.05		12.403	<0.0001
						CGRP of spinal cord	−0.015	0.003	−0.556	−5.398	<0.0001
AWR2	X is CGRP of spinal cord	0.627	0.394	0.384	Y = 1.069-0.023X	(Constant)	1.069	0.063		16.993	<0.0001
						CGRP of spinal cord	−0.023	0.003	−0.627	−6.494	<0.0001
AWR3	X is CGRP of spinal cord	0.657	0.432	0.423	Y = 1.48-0.03X	(Constant)	1.48	0.076		19.557	<0.0001
						CGRP of spinal cord	−0.03	0.004	−0.657	−7.034	<0.0001
AWR4	X is CGRP of spinal cord	0.633	0.4	0.391	Y = 1.73-0.03X	(Constant)	1.73	0.082		21.21	<0.0001
						CGRP of spinal cord	−0.03	0.005	−0.633	−6.585	<0.0001
AWR3	X is 5-HT of spinal cord	0.333	0.111	0.098	Y = 1.244-0.002X	(Constant)	1.244	0.1		12.461	<0.0001
						5-HT of spinal cord	−0.002	0.001	−0.333	−2.851	0.006
AWR4	X is 5-HT of spinal cord	0.295	0.087	0.073	Y = 1.47-0.001X	(Constant)	1.47	0.106		13.853	<0.0001
						5-HT of spinal cord	−0.001	0.001	−0.295	−2.49	0.015

3. Total Variance Explained and factor analysis

On the basis of the correlation matrix, a principle component analysis was carried out to examine. The results showed that total variance explained five characteristic value were >1, with a cumulative dedication rate at 76.48 %. The least cumulative dedication rate was 81.6 %. Factor significance was f1 > f2 > f3 > f4 > f5 > f6 . With component matrix = ±0.5 as weight, CGRP of spinal cord, CRD measurement, fecal pellets, sacchar-intake amount, CGRP of hypothalamus, CGRP of distal colon, 5-HT of spinal cord, NPY of hypothalamus were significant in the f1 factors.

KMO and Bartlett's test showed that Kaiser-Meyer-Olkin measure of sampling adequacy (KMO) at 0.804, and Bartlett's test of sphericity approx Chi-Square at 834.209 (P < 0.01), indicating the suitability of the factor analysis. Three factors F1, F2 and F3 (F1 > F2 > F3 in importance) were chosen for a rotated component matrix. F1 had the highest factor loading in CRD measurement (based on AWR Level), body weight and CGRP content of spinal cord. F2 was important in fecal pellet number; 5-HT content of colon, hypothalamus and spinal cord, CGRP content of hypothalamus and colon. F3 was important in the sacchar-intake, NPY content of colon and hypothalamus, CGRP and 5-HT content of spinal cord.

## Discussion

Psychological, character-related, genetic, contagious, and dietary factors all contribute to the etiology of IBS [6]. D-IBS patients typically have higher depression score than healthy control [7]. Food items that could induce D-IBS commonly include dairy products, chocolate, eggs and wheat, and more than 50 % patients of D-IBS have a history of food allergy [8]. In the current study, D-IBS was simulated using two methods (stress with vs. without senna decotion). The two methods produced comparable changes in colonic motility (increased fecal pellets), anhedonia (decreased sacchar-intake) and colonic visceral sensation (decreased CRD measurement). Immunologic pathology of the colon showed no change.

The two paradigms increased the 5-HT and CGRP, and decreased NPY content in the hypothalamus to similar degrees. Animal behaviors did not differ significantly between the CAS and ISC rats. EA treatment alleviated the IBS-like symptoms, and attenuated the changes of 5-HT, CGRP and NPY in BGA to a level comparable to the control.

5-HT is a major transmitter in BGA. 5-HT receptor antagonists have been used to manage D-IBS [9], and have been shown to reduce gastrointestinal motility and viscera sensitivity [10]. 5-HT receptor antagonists in combination with agents that target other nuerotransmitters in BGA are a promising approach [11]. CGRP and its receptors are enriched in dorsal root ganglion (DRG), and correlate with visceral hypersensitivity. NPY is a major neurotransmitter in the enteric plexus, and could affect cholinergic transmission in intestine mucous membrane inferior ganglion and regulate stress and mood by affecting hippocampus and hypothalamus.

In the current study, the correlation regression equations (Table 1) and linear relation on neurotransmitters in different BGA tissues showed that 5-HT but not CGRP and NPY content in the BGA was linearly correlated to IBS-like symptoms. However, via the action of 5-HT, both CGRP and NPY exhibited synchronous changes in hypothalamus and colon. The content of 5-HT, CGRP and NPY was correlated among each other in the colon, but not in the hypothalamus and spinal cord. The 5-HT and CGRP content in the same tissue was linearly correlated, but not correlated across hypothalamus, spinal cord and colon. Finally, the changes of 5-HT and CGRP in spinal cord was closely correlated to IBS-like symptoms and NPY in hypothalamus and colon, suggesting the 5-HT and CGRP in the spinal cord are important in the development of IBS.

The spinal cord is the relay station between the brain and gut, and thus critical for the development of IBS mechanism. Our findings support the hypothesis that the spinal cord is over-active in D-IBS. This could partly explain the apparent therapeutic action of EA. The exact mechanisms of action, however, need to be further analyzed.

## Conclusions

The two models (ISC vs. CAS) produced comparable IBS-like symptoms and changes in the BGA in rats. EA treatment could alleviate IBS-like symptoms, encouraging it clinical use in patients with D-IBS. The results also suggest that alteration of 5-HT in the brain, spinal cord and colon is most likely a major contributor to D-IBS. CGRP and NPY affects IBS development possibly via indirect action through 5-HT.
